# Non-COVID-19 deaths in times of pandemic

**DOI:** 10.1093/pubmed/fdac115

**Published:** 2022-11-14

**Authors:** Adan Silverio-Murillo, Jose Roberto Balmori de la Miyar, Alejandra Martínez-Alfaro

**Affiliations:** School of Government, Tecnologico de Monterrey; Business and Economics School, Universidad Anáhuac México; School of Government, Tecnologico de Monterrey

**Keywords:** COVID-19, health impact assessment, Mexico, mortality, non-COVID-19 deaths

## Abstract

**Background and objective:**

To investigate the effect of the COVID-19 pandemic on non-COVID-19 deaths in Mexico.

**Methods:**

This study analyzes monthly administrative data on 15 different causes of death in Mexico from 2017 to 2020. The effects of the COVID-19 pandemic on non-COVID-19 deaths are conducted using a difference-in-differences methodology and an event study.

**Results:**

The evidence shows mixed results. There is an increase in six causes of death: diabetes (36.8%), hypertension (25.8%), heart attacks (40.9%), bronchitis- asthma (24.2%), anemia (28.6%) and prostate cancer (21.4%). There is a decrease in two causes of death: traffic accidents (8.8%) and HIV (13.8%). There are null effects for seven causes of death: breast cancer, cerebrovascular disease, malnutrition, alcohol-related liver disease, renal insufficiency, homicides and suicides.

**Conclusions:**

The COVID-19 pandemic affected non-COVID-19 deaths caused by diseases that require intensive healthcare services. Conversely, this pandemic reduced social interactions, which contributed to a decrease on deaths such as traffic accidents.

## Introduction

Have the COVID-19 pandemic affected non-COVID-19 deaths? The decrease on health care utilization, the introduction of stay-at-home orders and the enforcement of social distancing measures may have contributed to both an increase and a decrease of non-COVID-19 deaths. That is, depending on the cause of non-COVID-19 deaths, the COVID-19 pandemic may have had a positive or negative effect.

On one hand, many patients with a non-COVID-19 disease, knowingly or unknowingly, have affected their care either by the decline in routine diagnostics or by the delay in treatment due to the prioritization of the most urgent diseases during the pandemic. For instance, the United States has seen an important decline on total admissions of patients with cardiovascular conditions during the pandemic^1^; this has eventually led to an increase in deaths related to ischemic heart disease, nationwide.^2^ Similarly, Peru has experienced an increase in suicide rates for men after the establishment of lockdowns^3^; this is associated to a deterioration in mental health after the onset of the COVID-19 pandemic.^4–9^

On the other hand, the pandemic has also changed the behavior of individuals, and thus their exposure to other infectious diseases and risky activities. For example, during the pandemic, Norway has seen 430 fewer deaths from causes other than COVID-19, much attributed to the reduced spread of other infectious diseases, to a decrease on transportation use, and to a decline on the use of hospitals that led to fewer individuals at risk of infections.^10^ Furthermore, Peru reported a significant decrease in deaths related to traffic accidents during the first part of the pandemic.^3^

This study analyzes the effect of the COVID-19 pandemic on non-COVID-19 deaths using administrative data from Mexico, including deaths attributed to heart attacks, diabetes, cerebrovascular disease, homicides, traffic accidents, suicides, among others. Based on the existing evidence, we hypothesize heterogeneous effects of the pandemic on non-COVID-19 deaths.

## Data and Methods

### Data

This study employs administrative data on mortality, collected by the National Statistics Office (INEGI). These administrative records are gathered from the States’ civil registrars, through the completion of a death certificate filled by a medical examiner. The data follow a series of validations to ensure its quality and to comply with the regulations regarding information on mortality in Mexico.^11^ This dataset contains information about causes, sex, age, date and place of death. The 2017-version of the death certificate continues to be valid throughout the period of analysis of this paper. In this certificate, there is only one main cause of death. Furthermore, there is information about pre-existing conditions that might have aggravated the main cause of death.

INEGI did verify that positively tested deceased patients were classified as COVID-19-related deaths. Most importantly, INEGI attributes a death to COVID-19 complications even if the deceased patient was a suspect case without a positive test. INEGI’s classification process, thus, tries to narrow down measurement error biases due to a lack of COVID-19 testing. Notwithstanding that INEGI’s classification process of causes of mortality is the most accurate in the country, we cannot rule out death-cause misclassification. According to actuarial estimations from INEGI, Mexico expected a total of 749 496 deaths in 2020. However, 1 076 417 deaths occurred that year. Thus, excess mortality is estimated at 326 921, out of which 200 256 excess deaths are attributed to COVID-19 complications, and 126 665, to other causes of mortality.^12^

For the analysis, we use monthly deaths per 100 000 inhabitants at the state level for 15 causes of mortality from the period 2017 to 2020. The selected causes of death are heart attacks, diabetes, cerebrovascular disease, hypertension, renal insufficiency, alcohol-related liver disease, malnutrition, breast cancer, prostate cancer, HIV, bronchitis-asthma, anemia, homicides, traffic accidents and suicides. By 2019, these 15 causes of mortality represent more than half of all deaths in Mexico. The total number of observations in the final sample is 1536 (4 years × 12 months × 32 states).

## Methods

**Difference-in-differences:** A difference-in-differences methodology is used to estimate the effects of the COVID-19 pandemic on non-COVID-19 deaths. This empirical strategy differentiates the pre-post change in the period affected by the COVID-19 pandemic (treatment) with the pre-post change in a non-pandemic period (control). We use the month interval 2020 m1–2020 m12 as the treatment group and 2017 m1–2019m12 as the control. In addition, we use m1–m2 as the pre-period and m3–m12 as the post-period. Employing this information, the effect is estimated as:


(1) \begin{eqnarray*} {\hat{\beta}}^{DD} =& \left({x}_{2020,m3\;\text{to}\;2020,m12}^{- post}-{x}_{2020,m1\;\text{to}\;2020,m2}^{- pre}\right)\notag\\ & -\left({x}_{2017-2019,m3\;\text{to}\;m12}^{post}-{x}_{2017-2019,m1\;\text{to}\;m2}^{pre}\right) \end{eqnarray*}

This difference-in-differences methodology assumes that the treatment and control groups follow a similar trend before the pandemic (parallel trends assumption), and shows the *average* impact of the months followed by the pandemic.

**Event study:** An event study is used to complement the difference-in-differences estimations. The event study provides the month-by-month impact before and after the pandemic. Thus, the event study allows us to check the parallel trend assumption using the month-by-month impact information before the pandemic, and presents the monthly dynamic effects after the onset of the pandemic and not only the average effect. In the event study, these outcomes can be visually observed by examining the dynamic of the effects before and after the pandemic.^13^ The econometric specifications used to estimate the difference-in-differences and the event study are presented in the Appendix.

## Results

### Descriptive statistics

Table 1 presents monthly deaths per 100 000 inhabitants at the state level from 2017 through 2020, divided by natural and non-natural causes of death. In the group of natural causes of death, the highest rates correspond to heart attacks (7.2), diabetes (7.1) and cerebrovascular disease (2.2). Conversely, the lowest rates of natural causes of deaths correspond to HIV (0.35), bronchitis and asthma (0.3), and anemia (0.2). In the group of non-natural causes of death, the following rates are observed: homicides (2.4), traffic accidents (1.1) and suicides (0.5).

**Table 1:** Descriptive statistics (monthly rate per 100 000 persons)

	Mean	SD	Min	Max
*Natural*				
Heart attack	7.24	2.92	2.10	28.21
Diabetes	7.08	2.76	2.40	27.49
Cerebrovascular disease	2.28	0.59	0.52	4.30
Hypertension	1.68	0.70	0.13	7.63
Renal insufficiency	0.90	0.32	0.00	3.05
Alcohol-related liver disease	0.81	0.46	0.00	2.72
Malnutrition	0.49	0.29	0.00	1.95
Breast cancer	0.48	0.20	0.00	1.34
Prostate cancer	0.47	0.18	0.00	1.53
HIV	0.35	0.25	0.00	1.84
Bronchitis and asthma	0.30	0.17	0.00	1.18
Anemia	0.23	0.16	0.00	1.03
*Other non-natural*				
Homicides	2.44	2.07	0.00	16.18
Traffic accidents	1.12	0.50	0.00	3.27
Suicides	0.52	0.27	0.00	1.61
*N*	1536			

SOURCE: INEGI mortality microdata.

### Difference-in-differences findings

Table 2 presents the difference-in-differences results for the 15 outcomes of interest. The estimations represent the changes in monthly rates per 100 000 inhabitants. These results are also presented in terms of percentage changes.

**Table 2:** Difference-in-differences results

	Diabetes	Hypertension	Heart-attack	HIV	Anemia	Malnutrition	Breast cancer	Prostate cancer
	(1)	(2)	(3)	(4)	(5)	(6)	(7)	(8)
1(COVID-19)	2.74^*^^*^^*^	0.49^*^^*^^*^	3.13^*^^*^^*^	−0.05^*^^*^	0.06^*^^*^	0.04	−0.01	0.09^*^^*^^*^
	(0.31)	(0.07)	(0.26)	(0.02)	(0.02)	(0.03)	(0.03)	(0.02)
*N*	1536	1536	1536	1536	1536	1536	1536	1536
*_R_* ^2^	0.66	0.63	0.66	0.69	0.57	0.68	0.50	0.39
Mean treatment pre-COVID	7.44	1.90	7.64	0.36	0.21	0.48	0.49	0.42
COVID-19 percentage change	36.82%	25.78%	40.96%	−13.88%	28.57%	8.33%	−2.04%	21.42%
Baseline FE	X	X	X	X	X	X	X	X
	*Bronchitis-asthma*	*Cerebrovascular disease*	*Alcohol-related liver*	*Renal insufficiency*	*Traffic accidents*	*Homicides*	*Suicides*	
	*(9)*	*(10)*	*(11)*	*(12)*	*(13)*	*(14)*	*(15)*	
1(COVID-19)	0.08^*^^*^	0.02	−0.04	−0.04	−0.09^*^	−0.19	−0.03	
	(0.02)	(0.04)	(0.04)	(0.04)	(0.04)	(0.13)	(0.03)	
*N*	1536	1536	1536	1536	1536	1536	1536	
*_R_* ^2^	0.41	0.63	0.78	0.48	0.61	0.76	0.58	
Mean treatment pre-COVID	0.33	2.52	0.84	1.07	1.02	2.35	0.51	
COVID-19 percentage change	24.24%	0.79%	−4.76%	−3.73%	−8.82%	−8.08%	−5.88%	
Baseline FE	X	X	X	X	X	X	X	

SOURCE: INEGI mortality microdata.

NOTES: Baseline fixed effects are included at the state, month and year. Robust standard errors are clustered at the state level. Significance levels: ^*^P < 0.05, ^*^^*^P < 0.01, ^*^^*^^*^P < 0.001

In the case of natural causes of death, we observe heterogeneous effects. Findings suggest an increase above the expected number of natural deaths for seven outcomes as a consequence of the pandemic: diabetes (36.8%), hypertension (25.8%), heart attacks (40.9%), anemia (28.5%), prostate cancer (21.4%) and bronchitis-asthma (24.2%). Furthermore, estimations point to a decrease on HIV-related fatalities (−13.8%) during the pandemic. Finally, there are no effects of the COVID-19 pandemic on the following natural causes of death: breast cancer, malnutrition, cerebrovascular disease, alcohol-related liver diseases and renal insufficiency.

In the case of non-natural causes of death, there are no increases for any of the outcomes analyzed as a result of the pandemic. Namely, evidence suggest a decrease on deaths related to traffic accidents (−8.8%), and null effects on homicides and suicides.

To confirm the robustness of our findings, we conduct the following tests: (i) we replicate our results using age-standardized rates, and (ii) we reproduce our results dropping those states that report zero cases for a particular cause of death for 1 month. First, there might be demographic differences (age structure) at the state level driving our results. Comparing rates between periods and geographical areas can be more representative when considering differences in age structure through age-standardized rates.^14^ The age-standardized rate is calculated as follows: age-standardized rate = 0.266*xRate*_0*–*14_ + 0.256*xRate*_15*–*29_ + .221*xRate*_30*–*44_ + .194*xRate*_45*–*64_ + 0.062*xRate*_65+_. The weights are obtained from the State of Mexico (the largest state in terms of population) for January 2017. The results are presented in Supplementary Appendix Table A.1. There are slight variations in the magnitude of the estimated coefficients; hence, the main results continue to hold.

Second, Table 1 reports states with no cases for certain causes of deaths for 1 month. These values are mostly driven by the least populated states such as Aguascalientes, Baja California Sur, Colima and Tlaxcala. Thus, we run the main specification without including these states that report zero cases of deaths for any given month. The results are included in Supplementary Appendix Table A.2. Again, we observe slight changes in the magnitude of the calculated coefficients, meaning that the main findings are robust to these issues.

### Event study findings

Figures F.1 and F.2 present the event study results which show the evolution of the effects on non-COVID-19 deaths before and after the pandemic. The point estimates are presented on a monthly basis by connected solid lines. The dashed lines indicate the 95% confidence intervals around the point estimates.

**Fig. 1:**
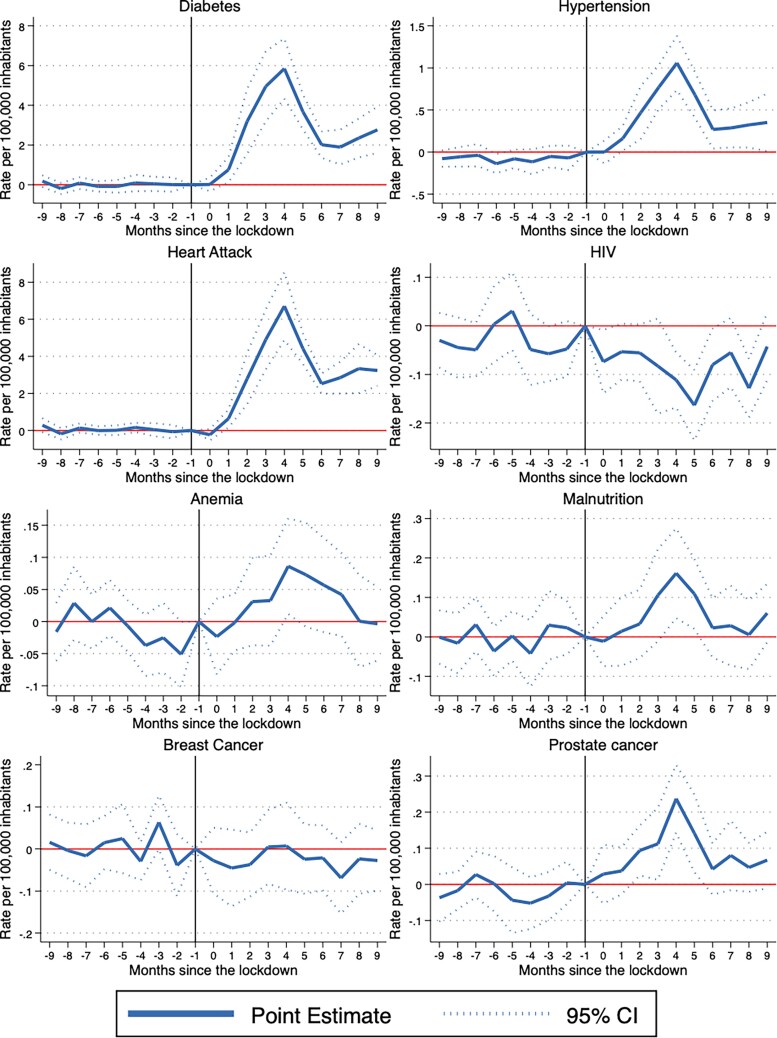
Event study results. S_OURCE_: INEGI mortality microdata. N_OTES_: Plotted coefficients are event study dummy variables, *β_q_*. Solid lines represent point estimates. Dotted lines display the 95% confidence intervals. Baseline fixed effects are included at the state, month and year. Robust standard errors are clustered at the state level.

**Fig. 2:**
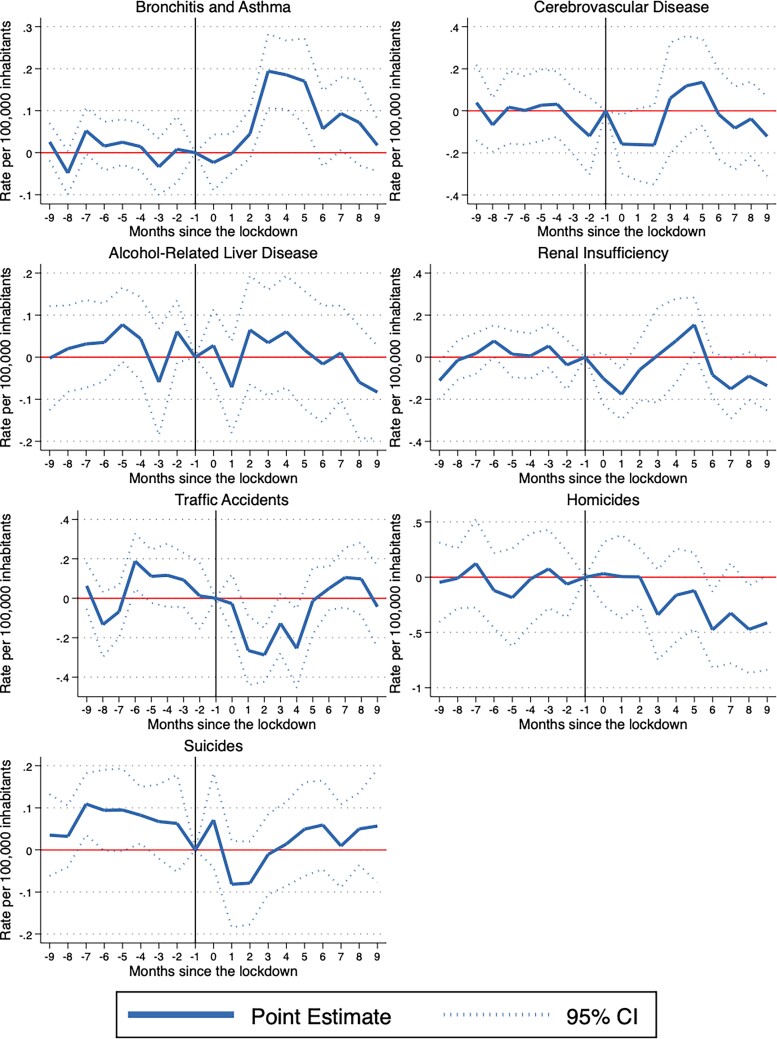
Event study results. S_OURCE_: INEGI mortality microdata. N_OTES_: Plotted coefficients are event study dummy variables, *β_q_*. Solid lines represent point estimates. Dotted lines display the 95% confidence intervals. Baseline fixed effects are included at the state, month and year. Robust standard errors are clustered at the state level.

All charts for every cause of death satisfy the parallel trend assumption, meaning that the treatment and control groups follow a similar trend before the pandemic. In general, the estimated coefficients prior to the start of the pandemic (period zero) are not statistically significant for the 15 outcomes of interest. Furthermore, the event studies show three different patterns for the eight variables that were statistically significant when using a difference-in-difference strategy. There are some causes of death that follow an inverted U-shape trend: Bronchitis-asthma, anemia and prostate cancer increase, and then return to pre-pandemic levels during the period of analysis. Other causes of deaths follow a U-shape trend. These are the cases of HIV and traffic accidents, which decrease and then return to pre-pandemic levels. Finally, there are some causes of deaths that increase but remain above pre-pandemic levels. The causes of death that follow this pattern are diabetes, hypertension and heart attacks.

It is possible that there are some effects not captured by the difference-in-differences strategy, but that can be observed using the event study. In particular, there are seven outcomes where the difference-in-differences suggest null effects. Yet, if these variables follow a U-shape trend, the difference-in-differences can give us an average impact close to zero. We analyze this potential situation for malnutrition, breast cancer, cerebrovascular disease, alcohol-related liver disease, renal insufficiency, homicides and suicides. In general, we observe that the event study results reflect the difference-in-differences findings. The only exception is malnutrition, for which the difference-in-differences point to null effects on average, even though the event study shows that this outcome follows an inverted U-shape trend.

## Discussion

### Main findings of this study

This paper analyzes the effects of the COVID-19 pandemic on non-COVID-19 deaths in Mexico. Using a difference-in-differences methodology and event study, the results show heterogeneous effects. Findings point to an increase on deaths related to diabetes, hypertension, heart attacks, bronchitis-asthma, anemia and prostate cancer, during the pandemic. Furthermore, there is a decrease on deaths related to traffic accidents and HIV after the onset of the pandemic. However, there are no effects on deaths attributed to breast cancer, cerebrovascular, malnutrition, alcohol-liver disease, renal insufficiency, homicides and suicides.

### What is already known in this topic

There is suggestive evidence of an important decrease of healthcare services in Mexico because of the COVID-19 pandemic.^15^ In particular, there is a significant decrease on healthcare services for chronic-degenerative diseases (42%), and health check-ups (50%) during the pandemic. The interruption of non-COVID-19 healthcare services during the pandemic could partially explain the increases observed in some non-COVID-19 deaths such as bronchitis-asthma and prostate cancer.^16^ Conversely, the pandemic and the lockdowns forced many individuals to stay at home, reducing traffic on the streets, and thus decreasing deaths related to traffic accidents. The findings for deaths attributed to traffic accidents in Mexico during the pandemic coincide with the existing evidence from Peru.^3^

Furthermore, the tremendous effects of the pandemic and lockdowns on mental health can potentially increase suicides. Evidence from Latin America suggests an important deterioration of individual’s mental health during the pandemic.^7^ In addition, findings from Mexico suggest an important decrease on mental health care utilization services of around 50% during the pandemic.^15^ However, despite these valid concerns, there is no evidence of an increase of suicides because of the pandemic. These results coincide with the trends of Google searches for words related to suicide, which did not peak during the pandemic in Latin American countries.^7^ The null effect on suicides can be partly explained by public health policies implemented during the pandemic by Mexico’s Ministry of Health. Namely, two important policies promoting mental health—launched during the course of the pandemic—are *Linea de la Vida* (Life Line) and *ConTacto*, both of which give free telehealth services through hotlines or messaging apps.^17^^,^^18^

In the case of HIV-related deaths, the disruption of antiretroviral drugs and the suspension of HIV testing in some countries pointed to an increase in the deaths attributed to HIV.^19^ However, individuals with HIV might be extra careful during a pandemic as to not put themselves at risk, which may decrease the number of deaths.^20^ In addition, physical distance measures might also lead to a decline in HIV contagion, given that risky sexual behaviors decreased.^19^ Evidence from a calibrated model for Baltimore (USA) suggests that a 25% reduction in sexual partners could reduce HIV infections by 12% over 1 year during the pandemic, whereas disruptions of antiretroviral drugs could only increase HIV-related deaths by 1.7% over the same time period.^21^ Also, other countries saw a rush to collect antiretroviral drugs before a planned suspension.^20^ The evidence from Mexico suggests that the factors that influence a decrease on HIV deaths overcome those related to an increase.

### What this study adds

This paper adds to literature by studying the case of a middle-income country, such as Mexico, which was particularly affected by the COVID-19 pandemic. It also contributes to the literature by using administrative data on mortality. Finally, this research is among the first to explore the impact of the pandemic on non-COVID-19 deaths by employing a difference-in-differences methodology and an event study.

### Limitations

A limitation of our data is that we cannot rule out death-cause misclassification; INEGI tried to classify certain deceased patients as COVID-19-related deaths, even if these were only suspects without an actual positive test. Another important limitation of this is that, due to the nature of the data, we cannot establish the mechanism by which COVID-19 increased these types of deaths. There are many mechanisms that potentially impacted the use of healthcare services during the pandemic, ranging from a decrease in the demand for health services due to fear of infection to a decrease in the supply of services due to redeployment of medical staff to care for COVID-19 patients. Future work can contribute to a better understanding of potential mechanisms as data become available. In a world where the population is likely to be impacted by new viruses and natural disasters, knowledge of these mechanisms can help policy makers to generate more effective policies to face these future public health challenges.

## Supplementary Material

Appendix_fdac115

## Notes

**Adan Silverio-Murillo**

**Jose Roberto Balmori de la Miyar**

**Alejandra Martínez-Alfaro**
