# Supplementary material for: Non-COVID-19 deaths in times of pandemic
Source: J Public Health (Oxf). 2022 Nov 14;45(2):e196–203. doi: 10.1093/pubmed/fdac115 (PMC10273375; doi:10.1093/pubmed/fdac115)
Supplement: Appendix_fdac115 [file appendix_fdac115.docx]

# Appendix

## Supplemental Methodology

The difference-in-differences effects are estimated as follows:

*Death_smy_* = *α* + *β* COVID-19*_smy_* + *a_s_* + *γ_m_* + *ν_y_* + *e_smy_* (2)

where *Death_smy_* is the outcome of interest for state *s* in month *m* and year *y*. COVID-19*_smy_* is a dummy variable that equals one from March through December, 2020. *a_s_* are state- fixed effects t. *γ_m_* are monthly-specific fixed-effects. *ν_y_* are year fixed effects. Standard errors are clustered at the state level.

The event-study specification is estimated as follows:

*Death_smy_* = *α* +

9

∑

*q*=*−*14

*q/*=*−*1

*β_q_*COVID-19*_sqy_* + *a_s_* + *γ_m_* + *ν_y_* + *e_sty_* (3)

where *Death_smy_* is the outcome of interest for state *s*, for month *m*, and year *y*. COVID-19*_sqy_* is a dummy variable that takes the value of one for each month *q* for state *s* before and after the start of the lockdown in March 2020. In particular, March 2020 is represented

by *q* = 0. *q* = *−*14 corresponds to fourteen months before the lockdown or January 2019. *q* = 9 represents nine months after the lockdown, or December 2020. Thus, the dynamic of the effects before and after the start of the pandemic in March 2020 are represented by the *β_q_* coefficients. The rest of the variables follow the same interpretation as in the difference-in-differences specification.

## Robustness Checks

Table A.1: Difference-in-differences Results: Age-standardized Rates

Cancer

|  | Diabetes |  | Hypertension |  | Heart- Attack |  | HIV |  | Anemia |  | Malnutrition |  | Breast Cancer |  | Prostate |  |
| --- | --- | --- | --- | --- | --- | --- | --- | --- | --- | --- | --- | --- | --- | --- | --- | --- |
|  | (1) |  | (2) |  | (3) |  | (4) |  | (5) |  | (6) |  | (7) |  | (8) |  |
| 1(COVID-19) | 2.51*** |  | 0.44*** |  | 2.77*** |  | -0.06** |  | 0.05** |  | 0.03 |  | -0.01 |  | 0.08*** |  |
|  | (0.28) |  | (0.06) |  | (0.23) |  | (0.02) |  | (0.01) |  | (0.03) |  | (0.03) |  | (0.02) |  |
| N | 1536 |  | 1536 |  | 1536 |  | 1536 |  | 1536 |  | 1536 |  | 1536 |  | 1536 |  |
| *R*2 | 0.63 |  | 0.54 |  | 0.56 |  | 0.68 |  | 0.51 |  | 0.63 |  | 0.39 |  | 0.24 |  |
| Baseline FE | X | X | | X | | X | | X | | X | | X | | X | | |
|  | Bronchitis- Asthma |  | Cerebrovascular Disease |  | Alcohol-Related Liver |  | Renal Insufficiency |  | Traffic Accidents |  | Homicides |  | Suicides |  | | |
|  | (9) |  | (10) |  | (11) |  | (12) |  | (13) |  | (14) |  | (15) |  | | |
| 1(COVID-19) | 0.07** |  | 0.01 |  | -0.03 |  | -0.03 |  | -0.10* |  | -0.18 |  | -0.04 |  | | |
|  | (0.02) |  | (0.04) |  | (0.04) |  | (0.04) |  | (0.04) |  | (0.13) |  | (0.03) |  | | |
| N | 1536 |  | 1536 |  | 1536 |  | 1536 |  | 1536 |  | 1536 |  | 1536 |  | | |
| *R*2 0.37 | | 0.47 | | 0.77 | | 0.45 | | 0.61 | | 0.75 | | 0.58 | | | | |
| Baseline FE | X | X | | X | | X | | X | | X | | X | | | | |

SOURCE: INEGI mortality microdata.

NOTES: Baseline fixed effects are included at the state, month, and year. Robust standard errors are clustered at the state level. Significance levels: * *p <* 0.05, ** *p <* 0.01, *** *p <* 0.001

Table A.2: Difference-in-differences Results: Not Including Zeros

Cancer

|  | Diabetes |  | Hypertension |  | Heart- Attack |  | HIV |  | Anemia |  | Malnutrition |  | Breast Cancer |  | Prostate |  |
| --- | --- | --- | --- | --- | --- | --- | --- | --- | --- | --- | --- | --- | --- | --- | --- | --- |
|  | (1) |  | (2) |  | (3) |  | (4) |  | (5) |  | (6) |  | (7) |  | (8) |  |
| 1(COVID-19) | 2.74*** |  | 0.49*** |  | 3.13*** |  | -0.05** |  | 0.06** |  | 0.05 |  | -0.01 |  | 0.09*** |  |
|  | (0.31) |  | (0.07) |  | (0.26) |  | (0.02) |  | (0.02) |  | (0.03) |  | (0.03) |  | (0.02) |  |
| N | 1536 |  | 1536 |  | 1536 |  | 1494 |  | 1465 |  | 1525 |  | 1535 |  | 1531 |  |
| *R*2 | 0.66 |  | 0.63 |  | 0.66 |  | 0.69 |  | 0.57 |  | 0.68 |  | 0.50 |  | 0.39 |  |
| Baseline FE | X | X | | X | | X | | X | | X | | X | | X | | |
|  | Bronchitis- Asthma |  | Cerebrovascular Disease |  | Alcohol-Related Liver |  | Renal Insufficiency |  | Traffic Accidents |  | Homicides |  | Suicides |  | | |
|  | (9) |  | (10) |  | (11) |  | (12) |  | (13) |  | (14) |  | (15) |  | | |
| 1(COVID-19) | 0.08** |  | 0.02 |  | -0.03 |  | -0.03 |  | -0.08† |  | -0.19 |  | -0.02 |  | | |
|  | (0.02) |  | (0.04) |  | (0.04) |  | (0.03) |  | (0.05) |  | (0.13) |  | (0.03) |  | | |
| N | 1498 |  | 1536 |  | 1532 |  | 1534 |  | 1534 |  | 1535 |  | 1529 |  | | |
| *R*2 0.41 | | 0.63 | | 0.78 | | 0.48 | | 0.62 | | 0.76 | | 0.60 | | | | |
| Baseline FE | X | X | | X | | X | | X | | X | | X | | | | |

SOURCE: INEGI mortality microdata.

NOTES: Baseline fixed effects are included at the state, month, and year. Robust standard errors are clustered at the state level. Significance levels: †*p <* 0.10, * *p <* 0.05, ** *p <* 0.01, *** *p <* 0.001
